# The adaptive immune system promotes initiation of prostate carcinogenesis in a human c-Myc transgenic mouse model

**DOI:** 10.18632/oncotarget.21305

**Published:** 2017-09-28

**Authors:** Monique H.M. Melis, Ekaterina Nevedomskaya, Johan van Burgsteden, Bianca Cioni, Hester J.T. van Zeeburg, Ji-Ying Song, John Zevenhoven, Lukas J.A.C Hawinkels, Karin E. de Visser, Andries M. Bergman

**Affiliations:** ^1^ Division of Molecular Genetics, Netherlands Cancer Institute, The Netherlands; ^2^ Division of Experimental Animal Pathology, Netherlands Cancer Institute, The Netherlands; ^3^ Division of Gastroenterology-Hepatology and Molecular Cell biology, Leiden university medical center, (LUMC), Netherlands; ^4^ Division of Immunology, Netherlands Cancer Institute, The Netherlands; ^5^ Division of Medical Oncology, Netherlands Cancer Institute, The Netherlands

**Keywords:** prostate cancer, GEMM, adaptive immune system

## Abstract

Increasing evidence from epidemiological and pathological studies suggests a role of the immune system in the initiation and progression of multiple cancers, including prostate cancer. Reports on the contribution of the adaptive immune system are contradictive, since both suppression and acceleration of disease development have been reported. This study addresses the functional role of lymphocytes in prostate cancer development using a genetically engineered mouse model (GEMM) of human c-Myc driven prostate cancer (Hi-Myc mice) combined with B and T cell deficiency (RAG1^-/-^ mice). From a pre-cancerous stage on, Hi-Myc mice showed higher accumulation of immune cells in their prostates then wild-type mice, of which macrophages were the most abundant. The onset of invasive adenocarcinoma was delayed in Hi-MycRAG1^-/-^ compared to Hi-Myc mice and associated with decreased infiltration of leukocytes into the prostate. In addition, lower levels of the cytokines CXCL2, CCL5 and TGF-β1 were detected in Hi-MycRAG1^-/-^ compared to Hi-Myc mouse prostates. These results from a GEMM of prostate cancer provide new insights into the promoting role of the adaptive immune system in prostate cancer development. Our findings indicate that the endogenous adaptive immune system does not protect against de novo prostate carcinogenesis in Hi-Myc transgenic mice, but rather accelerates the formation of invasive adenocarcinomas. This may have implications for the development of novel treatment strategies.

## INTRODUCTION

Prostate cancer is the second most common malignancy in men in the Western world [1]. The incidence of prostate cancer is rising due to more frequent screening and increasing life expectancy of men [2, 3]. The pathophysiological mechanisms of prostate cancer development are still poorly understood. It is well accepted that components of the microenvironment play a role in prostate carcinogenesis. The tumor-microenvironment consists of fibroblasts, infiltrating immune cells, endothelial cells, blood/lymph vessels and soluble factors like chemokines and cytokines. These host cells and mediators may affect cancer initiation and progression and even determine the prostate cancer cell of origin [4, 5]. Infiltrating immune cells are of particular interest since there is abundant evidence that inflammation plays a role in malignant transformation of multiple organs, including the prostate [6-9]. Both innate immune cells such as macrophages, and adaptive immune cells (T and B cells) have been reported to accumulate in the prostate throughout carcinogenesis [10, 11]. However, their contribution to prostate cancer development is contradictive, since both promotion and inhibition of carcinogenesis has been described [12, 13]. Therefore, their exact role in prostate cancer remains to be elucidated.

Genetically engineered mouse models (GEMM) are indispensible to study the role of components of the tumor-microenvironment in prostate cancer development [14]. The c-MYC onco-protein is a transcription factor, which upregulation is a prevalent and early change in prostate cancer and therefore considered a critical oncogenic event [15]. The Hi-Myc mouse model expresses human c-MYC in the prostate and recapitulates human prostate cancer on a genetic level, since cancers harbor loss of the tumor suppressor *Nkx3.1* and upregulation of the serine/threonine kinase *Pim-1*, which are also prevalent in human prostate cancer [16]. Moreover, it aligns with a multistep carcinogenesis model with luminal cells as the likely cell-of-origin of adenocarcinoma which is shared with human prostate cancer [16-18]. Here we assessed the functional significance of the adaptive immune system in prostate carcinogenesis by introducing B and T cell deficiency in the Hi-Myc GEMM of human prostate cancer [16].

## RESULTS

### Increased immune cell infiltration during prostate carcinogenesis

**Figure 1:**
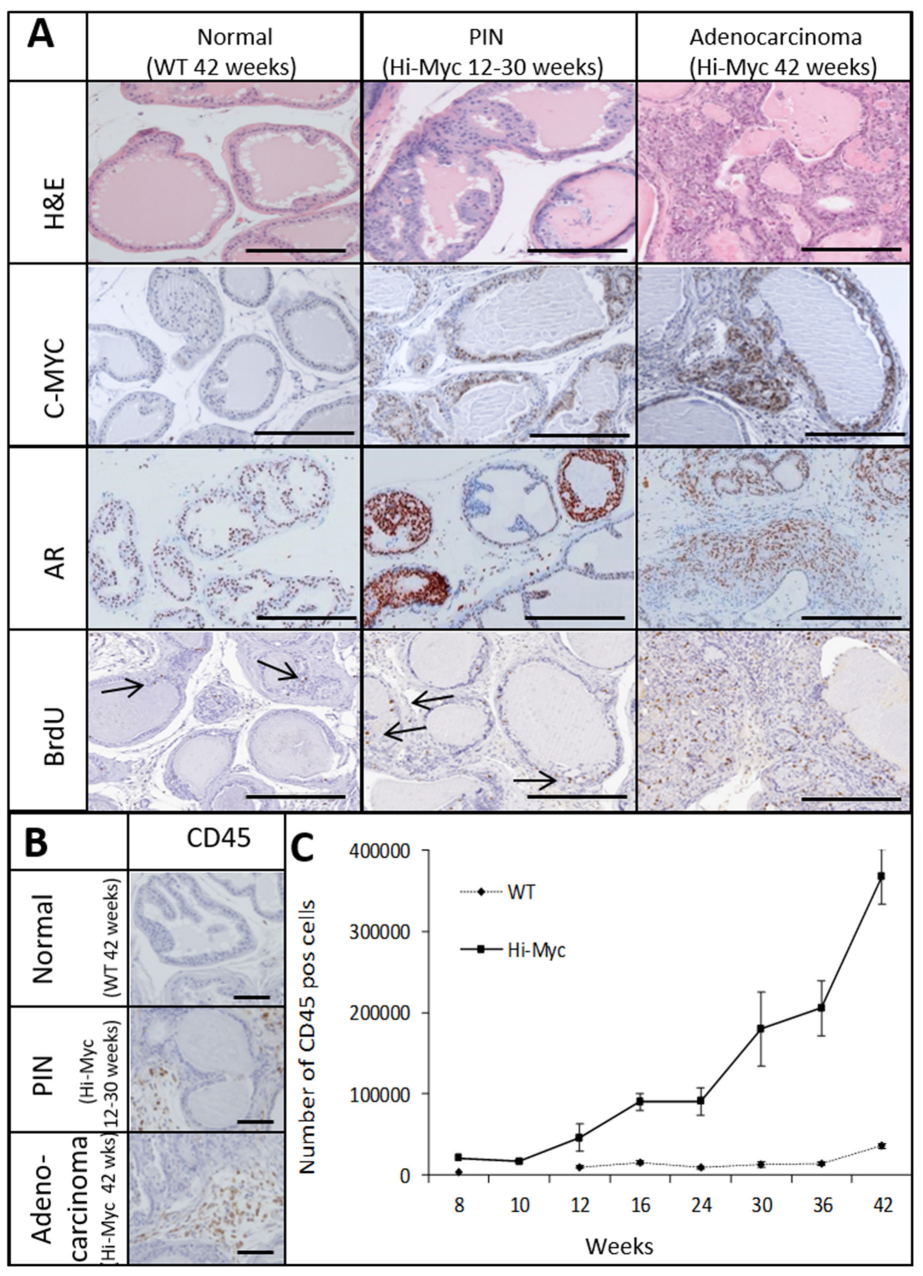
The Hi-Myc model follows a multistep prostate cancer development which is accompanied by an influx of immune cells Normal prostate tissue of Wild-Type (WT; 42 weeks) mice and PIN lesions (12-30 weeks) and Adenocarcinoma (42 weeks) of Hi-Myc mice. **(A)** H&E and IHC staining for C-MYC, Androgen Receptor (AR) and BrdU (bars =300uM, arrows indicate individual BrdU positive cells). **(B)** IHC staining for CD45 of WT (42 weeks) mouse prostates and PIN lesions (12-30 weeks) and Adenocarcinoma (42 weeks) in Hi-Myc mouse prostates. (bars =100μM) **(C)** Quantification of CD45 positive cells in Hi-Myc and WT mouse prostates by flow cytometry. A statistically significant higher number of CD45 positive cells was found in Hi-Myc mouse prostates compared to WT mouse prostates, from a precancerous age on (16-42 weeks, p<0.05, n=5, error bars are SEM).

Hi-Myc mice develop multistep prostate carcinogenesis. Prostate Intraepithelial Neoplasia (PIN) lesions were observed in mouse prostates from the age of 8 weeks on, while invasive adenocarcinomas were observed from the age of 30 weeks on. No pathological alterations were observed in control wild type (WT) mice at 42 weeks (Figure 1A). Overexpression of human c-MYC in Hi-Myc mouse prostates was confirmed by IHC (Figure 1A). Expression of the androgen receptor (AR), the single most important regulator of prostate cancer growth, was assessed throughout prostate carcinogenesis by IHC. Increased AR staining in PIN lesions was observed compared to normal prostate tissue (WT), but was partially lost in adenocarcinomas (Figure 1A). Increased nuclear BrdU incorporation as a measure of prostate cell proliferation rate was observed in PIN lesions and in adenocarcinoma compared to normal prostate tissue (Figure 1A).

The accumulation of immune cells throughout prostate carcinogenesis was assessed by IHC and flow cytometry for the common leukocyte marker CD45. CD45 positive cells were more abundant in Hi-Myc mouse prostates with precancerous PIN lesions (12-30 weeks) than in WT mouse prostates, which was further increased in invasive prostate cancer lesions (>30 weeks) (Figure 1B and 1C).

**Figure 2:**
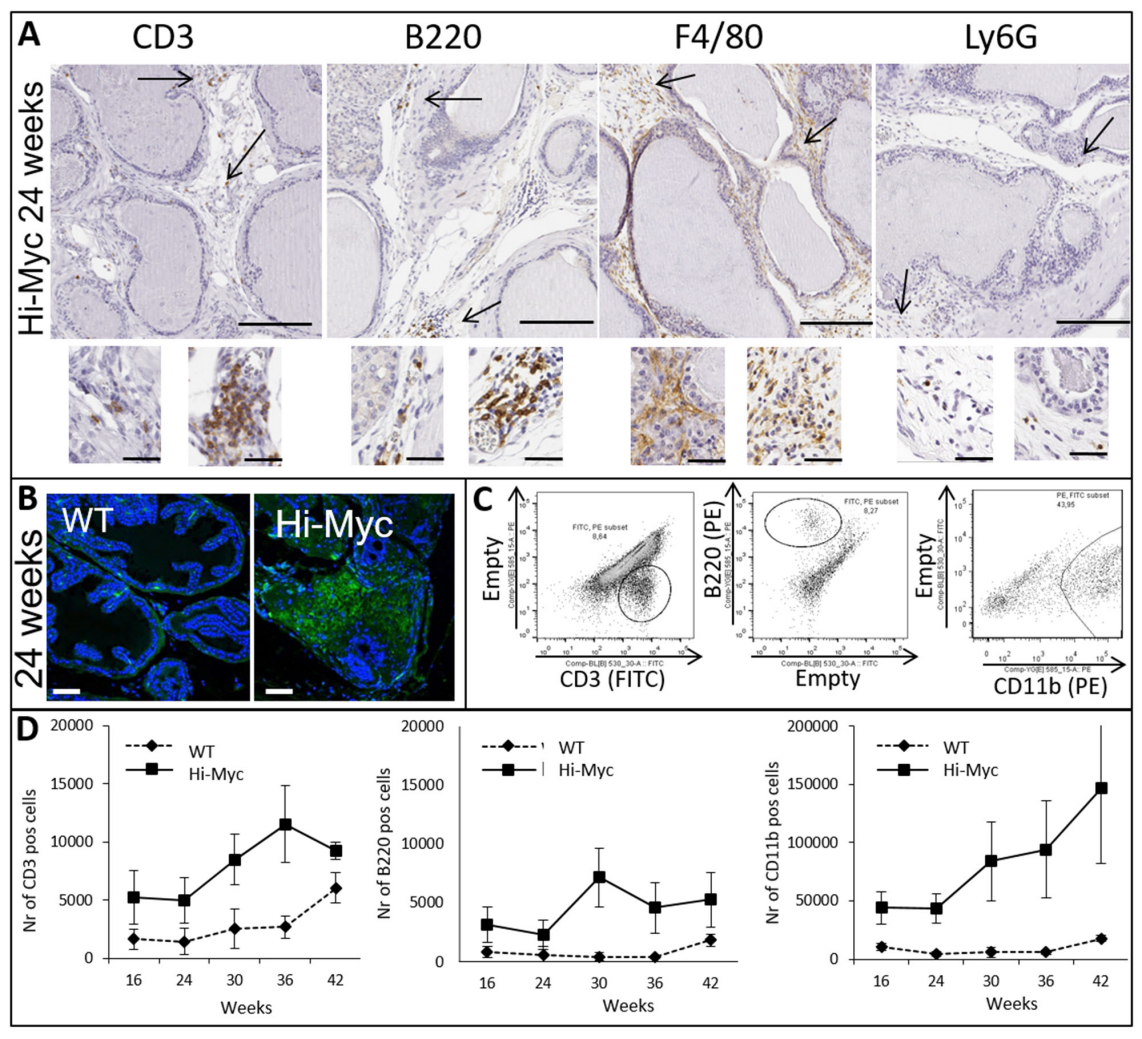
Influx of key players of the adaptive and innate immune system is observed in concert with prostate cancer development **(A)** IHC for CD3 (T cells), B220 (B cells), F4/80 (Macrophages) and Ly6G (Neutrophils) of precancerous Hi-Myc mouse prostates (24 weeks, bar = 200μM). Inserts are higher magnifications and show various organizations of cells within the prostate stroma (bar=50μM). **(B)** Immuno-fluorescence staining for IgG of WT and Hi-Myc mouse prostates (24 weeks, bar=75μM). **(C)** Flow cytometry; dot plots represent the CD3, B220 and CD11b/B220 positive cells in Hi-Myc mouse prostates after CD45 selection. **(D)** Quantification of CD3, CD19 and CD11b/B220 positive cells by flow cytometry. Higher numbers of CD3, CD19 and CD11b positive cells accumulated in Hi-Myc mouse prostates compared to WT mouse control prostates, from a precancerous age on (16-42 weeks, n=5, p<0.05, error bars are SEM).

Identification of the various immune cell populations (CD3^+^ T cells; CD19 or B220+ B cells; F4/80+ macrophages; and Ly6G+ neutrophils) in prostates at the precancerous age of 24 weeks was assessed by IHC (Figure 2A). While macrophages were dispersed throughout the stroma, B and T cells were found in clusters. Neutrophils were only observed sparsely in the stromal compartment throughout prostate carcinogenesis. Abundant IgG immunoglobulin depositions were observed in Hi-Myc compared to WT mouse prostates (Figure 2B). Flow cytometry was used to quantify the number of CD45 (a general marker in all leukocytes), CD3 (T-cells), B220 (B-cells) and CD11b (common myeloid cell marker, both for macrophages and neutrophils) positive cells (gating is shown in Figure 2C) in the prostates of 16 week old mice (pre-cancerous age) through invasive adenocarcinoma in 42 week old mice. Similar to the total number of leukocytes (Figure 1C), the number of T (CD3) and B (B220) cells and the amount of CD11b positive cells increased with age and prostate carcinogenesis (Figure 2D). F4/80+ macrophages were the most abundant infiltrating cells as suggested by IHC (Figure 2A).

### Genetic elimination of B and T cells attenuates prostate carcinogenesis in concert with delayed infiltration of other immune cells

In order to investigate the role of T and B cells in prostate cancer development, both adaptive immune cell types were genetically eliminated from Hi-Myc mice by crossing these mice with RAG1^-/-^ mice. Absence of T and B cells and IgG depositions was confirmed in Hi-MycRAG1^-/-^ and RAG1^-/-^ mice by IHC and/or flow cytometry (Supplementary Figure 1A, 1B and 1C).

**Figure 3:**
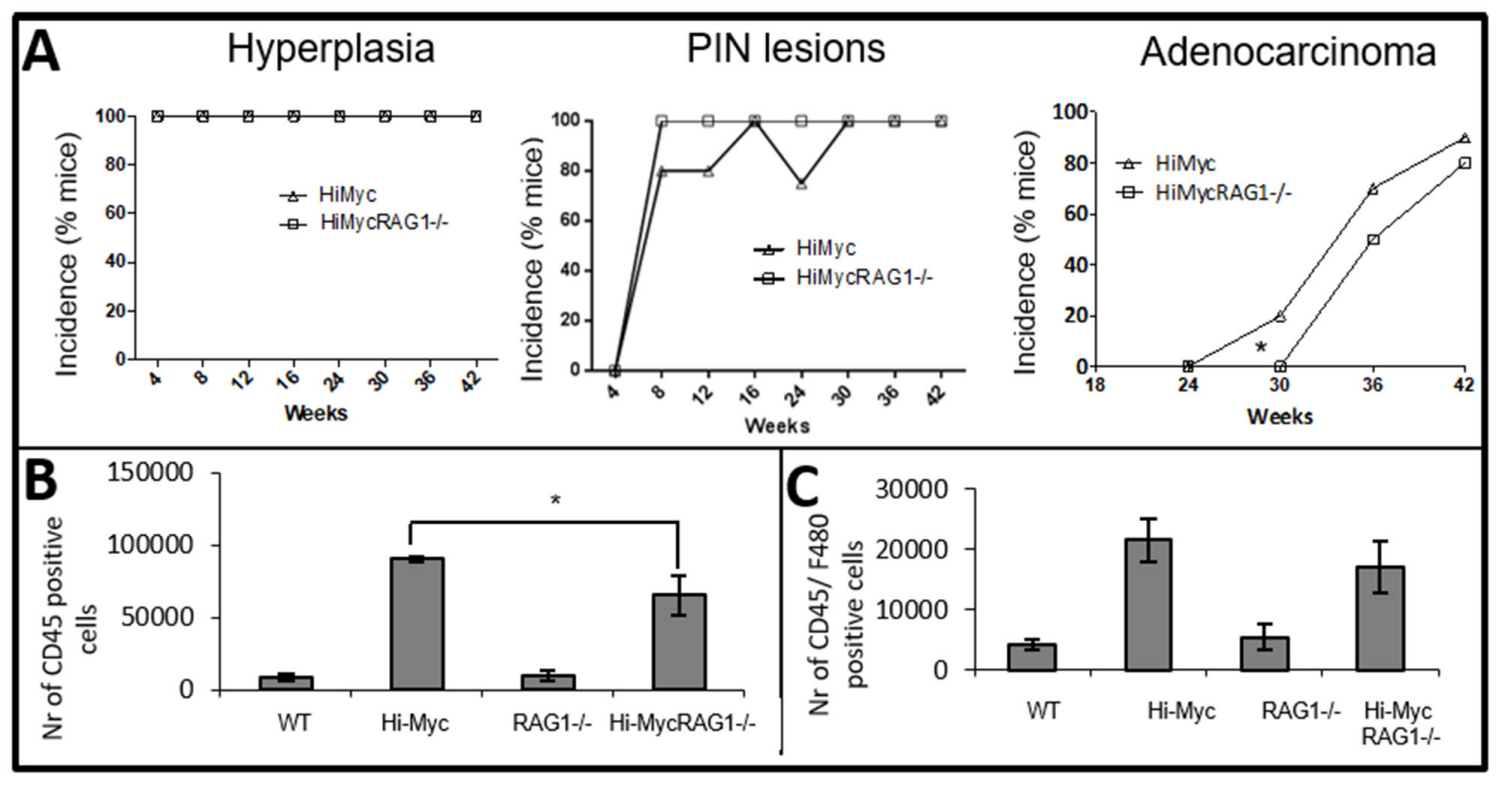
Reduced immune cell influx and delayed development of adenocarcinoma in Hi-MycRAG1^-/-^ compared to Hi-Myc mice **(A)** A pathologist evaluated prostates (blindly) of 4 to 42 weeks old mice for the development of prostate cancer and its preneoplastic stages (10 mice per genotype). Hyperplasia was observed from 4 weeks of age and PIN lesions from 8 weeks of age in Hi-Myc and Hi-MycRAG1^-/-^ mouse prostates. There was no difference in development of the preneoplastic stages between Hi-Myc and Hi-MycRAG1^-/-^ mouse prostates. However, onset of adenocarcinoma was delayed in Hi-MycRAG1^-/-^ mouse prostates (^*^p<0.05). **(B)** Quantification of CD45 positive cells in 24 weeks old mouse prostates was assessed by flow cytometry. Very low numbers were found in WT and RAG1^-/-^ mouse prostates. A statistically significant lower amount of CD45 positive cells accumulated in Hi-MycRAG1^-/-^ compared to Hi-Myc mouse prostates (n=5, ^*^p<0.05, error bars are SEM). **(C)** Quantification of F4/80 positive cells was assessed by flow cytometry. A trend was observed of less F4/80 positive cells in Hi-MycRAG1^-/-^ compared to Hi-Myc mouse prostates (n=5, error bars are SEM).

Next, the influence of the adaptive immune system on development of prostate cancer was studied. As shown for WT mice, no pathological alterations were observed in RAG1^-/-^ mouse prostates (Figure 1A, Supplementary Figure 2A, left panel). In Hi-MycRAG1^-/-^ and Hi-Myc mice, hyperplasia of the prostate was observed from the first time point analyzed, at 4 weeks of age (Figure 3A, Supplementary Figure 2A, right panel). Also PIN lesions were observed at early time points. Hi-MycRAG1^-/-^ and Hi-Myc mouse prostates contained PIN lesions from 8 weeks onwards (Figure 3A). Interestingly, a statistically significant delay in latency of invasive adenocarcinoma development was observed between Hi-MycRAG1^-/-^ and Hi-Myc mice (p<0.05) (Figure 3A). The earliest time point invasive adenocarcinomas were observed in Hi-Myc mouse prostates was at 30 weeks of age, while no invasive adenocarcinomas were observed in age matched Hi-MycRAG1^-/-^ mice (Figure 3A). In Hi-MycRAG1^-/-^ mouse prostates the first invasive adenocarcinomas were observed with a 6 weeks delay (Figure 3A). From that point no difference in the rate of adenocarcinoma development was observed, which suggested a difference in latency but no difference in rate of progression of prostate cancer. This small, but statistically significant delay in prostate cancer onset indicates that the adaptive immune system does not protect against de novo prostate carcinogenesis, but rather promotes prostate cancer initiation. No differences were observed in the morphology or invasive phenotype of adenocarcinoma in Hi-Myc or Hi-MycRAG1^-/-^ prostates (Supplementary Figure 2B).

We next evaluated whether the absence of lymphocytes altered the composition of innate immune cell populations in the prostate cancer microenvironment. Accumulation of CD45 and F4/80 positive cells in Hi-MycRAG1^-/-^ and Hi-Myc mouse prostates was quantified by flow cytometry at the pre-invasive cancer age of 24 weeks. A significantly lower amount of CD45 positive cells was found in Hi-MycRAG1^-/-^ compared to Hi-Myc mouse prostates (Figure 3B). Although not reaching statistical significance, lower numbers of F4/80 positive cells were found in Hi-MycRAG1^-/-^ compared to Hi-Myc mouse prostates at the pre-invasive cancer age of 24 weeks (Figure 3C).

### Altered cytokine expression profiles in the prostates of B and T cell depleted mice during carcinogenesis

**Figure 4:**
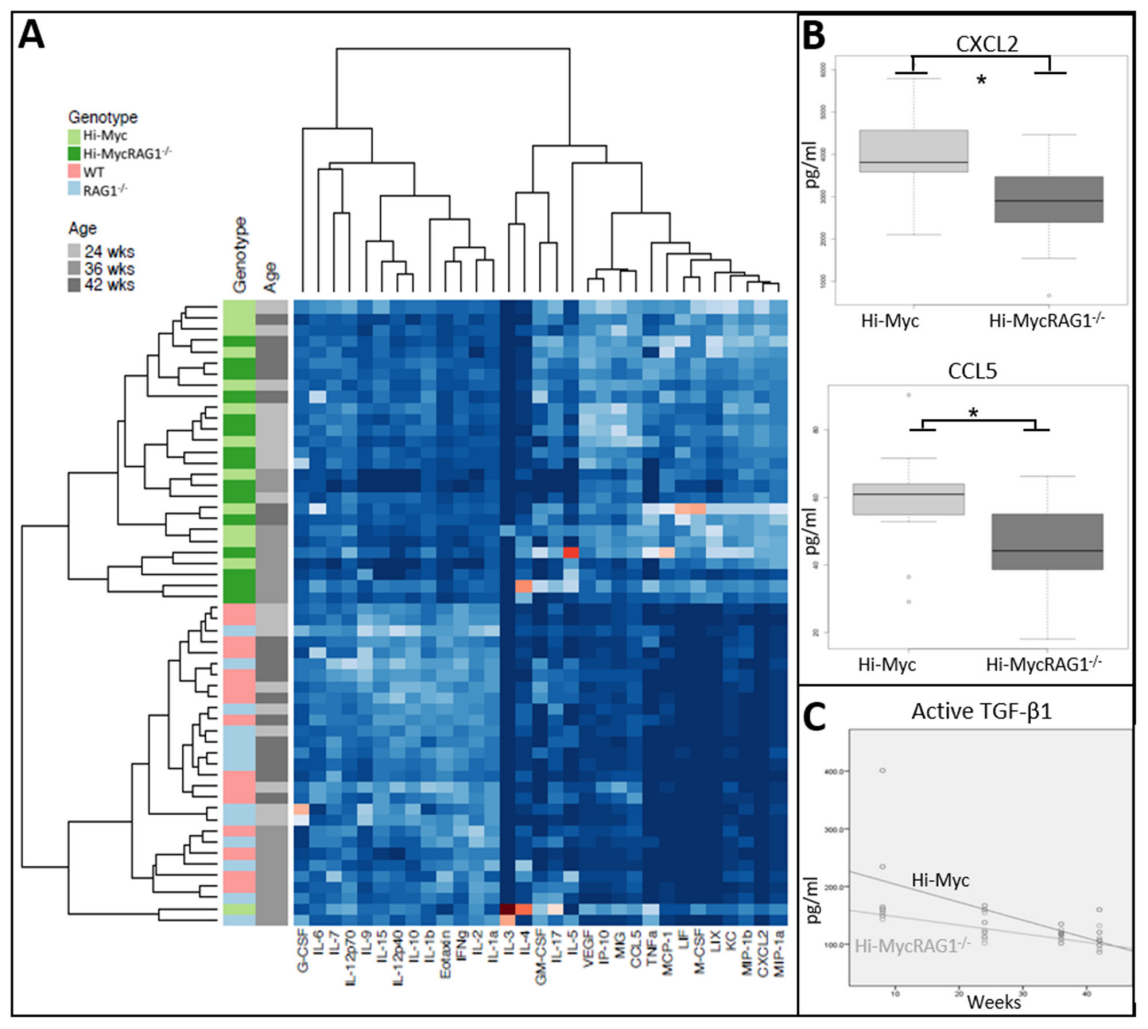
Absence of the adaptive immune system changes the cytokine profile in neoplastic prostate tissue **(A)** Unsupervised hierarchical clustering of the expression of 32 cytokines as assessed by Luminex array. Two distinct groups of cytokines were identified, which corresponded with normal prostate tissue (WT, RAG1^-/-^) and prostate cancer (Hi-Myc and Hi-MycRAG1^-/-^, 24- 42 weeks of age). **(B)** Expression levels for CXCL2 and CCL5 in Hi-Myc and Hi-MycRAG1^-/-^ mouse prostates as assessed by luminex array showed a statistically significant lower expression of both CXCL2 (^*^p<0.015) and CCL5 (^*^p<0.03) in Hi-MycRAG1^-/-^ compared to Hi-Myc mouse prostates (n=5, 24-42 weeks of age). **(C)** TGF-β1 levels in Hi-Myc and Hi-MycRAG1^-/-^ mouse prostates were measured by a DuoSet ELISA. The genotype, either Hi-Myc or Hi-MycRAG1^-/-^ was an independent predictor of the active TGF-β1 level. TGF-β1 levels were initially lower in Hi-MycRAG1^-/-^ compared to Hi-Myc mouse prostates (n=5, p<0.05).

As a next step, we assessed the levels of various inflammatory (chemotactic) mediators in the prostates of Hi-Myc, Hi-MycRAG1^-/-^, WT and RAG1^-/-^ mice. Levels of a total of 32 chemokines and cytokines were measured in whole prostate protein lysates using a Multiplex assay. Unsupervised clustering analysis showed a distinct cytokine profile in normal prostate (WT and RAG1^-/-^) and adenocarcinoma containing prostates (Hi-Myc, and Hi-MycRAG1^-/-^> 24 weeks of age) (Figure 4A). Subsequently, we explored whether T and B cells deficiency resulted in an altered cytokine profile during prostate cancer development. Statistically significant lower expression levels of CXCL2 (macrophage inflammation protein 2 (MIP2) and CCL5 (RANTES) were observed in Hi-MycRAG1^-/-^ compared to Hi-Myc mouse prostates from a pre-invasive adenocarcinoma age onwards. (>24 weeks of age) (Figure 4B; Supplementary Figure 3B). Both cytokines are described to be involved in the attraction of innate immune cells e.g. macrophages [19, 20]. This is in line with the observed lower numbers of leukocytes and suggested lower numbers of macrophages in prostates of Hi-MycRAG1^-/-^ mice compared to Hi-Myc mice (Figure 3B and 3C). Levels of lymphotoxin β and Transforming growth factor beta 1 (TGF-β1), two cytokines previously reported to be implicated in prostate cancer, were assessed in mouse prostates by ELISA. Lymphotoxin β transcript was not detectable in Hi-Myc or Hi-MycRAG1^-/-^ mouse prostates (data not shown). Total TGF-β1 showed a stepwise increase with stage of prostate carcinogenesis, but no statistically significant difference was observed between Hi-MycRAG1^-/-^ and Hi-Myc mouse prostates (Supplementary Figure 3C). Levels of active TGF-β1 however, were lower in Hi-MycRAG1^-/-^ than in Hi-Myc mouse prostates at the precancerous age of 8 weeks, while this difference disappeared at later time points, the genotype appeared to be an independent predictor (Figure 4C, p<0.05).

Next, we investigated expression of CXCL2, CCL5 by specific immune cells isolated from spleen, the prostate associated lumbar lymph nodes and the prostate microenvironment. CXCL2 was exclusively expressed by CD11b^+^ cells isolated from the prostate, while CCL5 was expressed by CD3^+^ and CD11b^+^ cells (Supplementary Figure 3B). No CXCL2 expression was detected in CD3^+^ and CD11b^+^ cells isolated from spleen and lumbar lymph nodes (data not shown).

In conclusion, these results suggest that less immune cells were attracted and less cytokines were released in the prostate microenvironment of Hi-MycRAG1^-/-^ mice compared to Hi-Myc mice which microenvironment could therefore be defined as less inflammatory.

## DISCUSSION

Despite some advances in the treatment of prostate cancer, new prognostic biomarkers and novel therapies remain urgently needed [1]. Inflammatory infiltrates of T and B cells are found in the prostate microenvironment throughout carcinogenesis, which might hold promise that immunotherapy is an effective treatment for prostate cancer patients [21-23]. However, to date immune checkpoint inhibitors did not establish survival benefit of prostate cancer patients [24]. This underlines the notion that the involvement of the adaptive immune system in prostate cancer is poorly understood [10, 11, 25, 26].

We observed delayed prostate cancer development in adaptive immune system deficient Hi-MycRAG1^-/-^ mice compared to immune-proficient Hi-Myc mice. Our data suggests that *de novo* prostate cancers in Hi-Myc mice do not elicit effective spontaneous anti-tumor T cell responses, but rather accelerate the formation of invasive adenocarcinoma. These findings are in line with a previous study from Lai et al, who described that the absence of T and B cells attenuated the formation of precancerous PIN lesions in a PTENF^+/-^ GEMM for prostate cancer [18]. However, their model is restricted to PIN lesions and the relation between these lesions and the development of invasive adenocarcinoma is unclear [27, 28]. Furthermore, our data is also supported by a study in the transgenic adenocarcinoma mouse prostate (TRAMP) model, which shows delayed prostate cancer in the absence of lymphocytes [29]. However, the TRAMP model develops neuroendocrine carcinomas instead of adenocarcinomas and therefore only models a fraction of primary human prostate cancers.

The number of infiltrating CD45 positive cells was higher in Hi-Myc mice than in WT mice at an age of 8 weeks. At 8 weeks, hyperplasia of the epithelium was found, which is not considered premalignant [30]. The accumulation of immune cells in this premalignant stage suggests a role of the infiltrating immune cells in prostate cancer initiation. During epithelial transformation, the numbers of infiltrating CD45 positive cells further increased which was also observed in mouse models of various other cancers [31].

Infiltrating immune cell populations were specified. T (CD3; both CD4 and CD8 although data not shown) and B lymphocytes and CD11b positive myeloid cells accumulated in the Hi-Myc mouse prostates in concert with cancer development. These increases in immune cell populations have also been described in other GEMM models of prostate cancer and throughout human prostate carcinogenesis [10, 11, 18]. In line with previous studies reporting that the adaptive immune system regulates the recruitment of innate immune cells to the tumor microenvironment [12, 22, 23, 32], we observed a reduction in the accumulation of CD45 positive cells and a non-statistically significant decrease in infiltrating macrophages in Hi-Myc prostates in the absence of T and B cells.

Soluble factors like chemokines and cytokines play a pivotal role in the recruitment and functions of immune cells in the tumor microenvironment [19, 33]. Prostate cancer development in the Hi-Myc mouse model was associated with a distinct cytokine profile. Absence of B and T cells was associated with decreased levels of TGF-β1, and reduced levels of CXCL2 and CCL5, both attractants of macrophages. In humans, both CXCL2 and CCL5 have been suggested to promote prostate cancer development and indeed increased CCL5 levels were observed in human prostate cancer [34-36]. Similar observations were made in human breast cancer in which CCL5, expressed by the tumor microenvironment, exerted tumor promoting activity by shifting the balance from an anti- to a pro-tumor microenvironment and inducing infiltration of macrophages with a cancer promoting phenotype [19, 20]. TGF-β is thought to enhance prostate cancer growth and metastasis by stimulating angiogenesis as well as inhibiting immune responses directed against tumor cells, depending on stage of disease [37, 38]. Various immune cell populations including lymphocytes and myeloid cells secrete TGF-β1, which can polarize many components of the immune system resulting in either anti or pro-tumor responses [39]. Total TGF-β1 was increased in mouse prostate cancer. In the absence of T and B cells, lower levels of active TGF-β1 were associated with reduced infiltration of immune cells and delayed prostate cancer development. Our findings are supported by the study of Wu et al, which reported that TGF-β1 is associated with recruitment of immune cells, resulting in a more immunosuppressive tumor microenvironment and a more aggressive prostate cancer [37, 38].

Although we cannot discriminate between the role of T and B cells in our model, both have been implicated in prostate carcinogenesis. Studies in the TRAMP mouse model of prostate cancer have suggested that B cell infiltration is required for prostate cancer progression and tumor recurrence whereas B cell secreted lymphotoxin β promoted castration resistant prostate cancer [26, 40]. In Hi-Myc mouse prostates, we were unable to detect lymphotoxin β, which might be due to the very low numbers of B cells in the Hi-Myc mouse prostates or the early stages of prostate cancer we analyzed.

As observed in our mouse model, an increased accumulation of B cells was reported in human prostate cancer compared to normal prostate tissue [41]. Treatment of prostate cancer patients with the B cell marker CD20 directed monoclonal antibody Rituximab resulted in some biomarker response, suggesting a role of B cells in prostate cancer growth [42]. There is also data supporting a role of T cells in the development of prostate cancer, which is based on epidemiological studies that showed a lower incidence of prostate cancer in patients with suppressed T cells as a result of an human immunodeficiency virus (HIV) infection [43].

Furthermore it was suggested that B and T cells instruct the microenvironment and innate immune cells towards a more pro-tumor state [22, 26, 44], which is in line with our findings in the Hi-Myc model that B and T cells promote prostate cancer development.

In conclusion, in the Hi-Myc GEMM of prostate cancer, carcinogenesis is associated with a steady accumulation of immune cells from a pre-invasive cancer stage onwards. Invasive prostate cancer was delayed in the absence of B and T lymphocytes. In the prostates of mice lacking the adaptive immune system, lower numbers of immune cells were found concurrent with lower levels of the cytokines CXCL2, CCL5 and TGF-β1. The absence of T and B cells delayed the formation of adenocarcinoma of the prostate in the Hi-Myc model. Further studies are needed to identify the exact subsets of immune cells that play a pivotal role in prostate carcinogenesis, which might have implications for development of novel strategies for effective anticancer treatment.

## MATERIALS AND METHODS

### Genotyping of mice

Animal experiments were approved by the Animal Ethics Committee of the Netherlands Cancer Institute and performed in accordance with institutional, national and European guidelines for Animal Care and Use. Hi-Myc mice (FVB-Tg (ARR2/Pbsn-MYC) 7Key) were obtained from the National Cancer Institute at Frederick, Mouse Repository. Generation of the Hi-Myc model was described previously [16]. Hi-Myc mice were intercrossed with RAG1^-/-^ mice (a generous gift from Lisa Coussens, now at Oregon Health and Science University, USA) to generate breeding colonies: Female Hi-Myc;RAG1^+/-^ mice were crossed with male RAG1^-/-^ to generate the 4 cohorts of genotypes (Hi-Myc;RAG1^+/-^ (Hi-Myc), Hi-Myc;RAG1^-/-^ (Hi-MycRAG1^-/-^), RAG^+/-^ (WT) or RAG1^-/-^ at equal ratios. Mice were on a FVB background (F>10), were kept in individually ventilated cages at the animal care facility of the NKI with food and water *ad libitum* and experimental groups contained 5 or more male mice. Genotyping of Hi-Myc and RAG1^-/-^ mice was performed by PCR analysis on ear DNA using the primers from Invitrogen as listed in Table 1. None of the genotypes had phenotypic alterations.

**Table 1:** Primer sequences for genotyping

Genotype	Primer	Sequence
RAG1^-/-^	Primer 1: RAG-4	5’-AGACACAACGGCTTGCAACACAG-3’
	Primer 2: RAG-5	5’-TGCCGAGAAAGTCCTTCTGCCAG-3’
	Primer 3: RAG-6	5’-GTGGAATGAGTGCGAGGCCAGA-3’
Hi-Myc	Primer 1: PBMY Forward	5’-ACCACCAGCAGCGACTC-3’
	Primer 2: PBMY Reverse	3’-TTC AGCTCGTTCCTCCTC-5’
Actin	Primer 3:b_actin_f	5’-TGTGACGTTGACATCCGTAA-3’
	Primer 4:b_actin_r	3’-TGCTAGGAGCCAGAGCAGTA-5’

### Histological analysis and Immunohistochemistry

For histological analysis, prostate tissues were collected from 4, 8, 12, 16, 24, 30, 36 and 42 weeks old mice of Hi-Myc, Hi-MycRAG1^-/-^ and their WT and RAG1^-/-^ controls, respectively (n=10 per genotype and per time point). The prostate tissues were fixed in 4% (vol/vol) formalin (Klinipath) and subsequently dehydrated and embedded in paraffin. Two μm thick paraffin sections were cut on 5-10 semi series levels, Haematoxylin and Eosin (H&E) stained, and were evaluated blindly for lesions such as hyperplasia, PIN, local micro-lesions of adenocarcinoma and invasive adenocarcinoma. For immunohistochemistry (IHC) studies, 4 μm thick sections were made, deparaffinated, rinsed and antigen retrieval was performed by incubating the slides for 20 min at 37°C in 20 ug/ml Proteinase K (Sigma Aldrich P6556 for Ly6G, F4/80, CD45), 30 min citrate buffer (BioGenex; HK086-5K for BrdU, B-220) or by 30 min in TRIS (Biosolve)/EDTA (Sigma) pH9.0 (for CD3, CD4, CD8) at 100°C. Endogenous peroxidase activity was inhibited by 20 min of incubation of 3% H_2_0_2_ (Sigma; A-31642) in methanol (Sigma). Sections were preincubated in 10% non-fat milk (Campina) for 30 min and incubated with antibodies of interest: c-MYC (Santa cruz, sc-40), BrdU (Dako, M0744), CD3 (Thermo Scientific SP7 RM 9107), CD4 (eBiosience, 14-9766-82), CD8 (eBiosience, 14-0808-82), B220 (BD Biosciences 557390), CD45 (BD Biosciences 553076.), Ly6-G (BD Biosciences 551459) and F4/80 (AbD Serotec MCA 497) O/N at 4°C in PBS/1% BSA (Gibco)/1.25% normal goat serum (Sanquin) and their appropriate secondary antibody. 3’-3 diaminobenzidine (Sigma D-5905) plus H_2_0_2_ or chromagen (Dako K3468) was used for visualization. Slides were counterstained with haematoxylin, dehydrated and mounted with Entellan. HE and IHC sections were digitally processed using the Aperio Scanscope and imaged using ImageScope software version 11.0.2.

### Confocal Analysis-Immunofluorescence staining

For immunofluorescence (IF) staining slides were prepared as described above for IHC and stained with anti-IgG antibody (1:50, Sigma F8264) as described [23]. Images were captured on a Leica SP5C Spectral Confocal Laser Scanning Microscope.

### Flow cytometry

Single cell suspensions of whole prostates were derived using a tissue-chopper (McIlwain) followed by 2 hours incubation in DMEM (Gibco) containing 1 mg/ml collagenase (Roche) plus hyaluronidase 5000 u/ml (Sigma) at 37°C. Samples were filtered, spun down and re-suspended in FACSbuffer (PBS +0.5% BSA), stained with antibodies against CD45, CD11b, CD3, CD19, B220, F4/80, Ly6G (APC, FITC and PE, all Ebioscience) and analyzed (whole single cell suspension sample for absolute numbers) on a FACS Fortessa (BD Biosciences) using FlowJo software (V10).

### Cytokine assays

Prostates were lysed in lysis buffer (5 μl per mg tissue Millipore, Amsterdam, NL) supplemented with 1:100 protease cocktail inhibitor (Sigma-Aldrich). Tissue was grinded in 1.5 ml micro centrifuge tubes using grinders (Eppendorf, cat no 0030 120.973A), followed by 1 hour incubating/rotating at 4°C. Next, samples were centrifuged and supernatant was used for Luminex cytokine profiling, 100 μg was used per sample. A Milliplex Mouse Cytokine/Chemokine Magnetic premixed Bead panel immunoassay (Millipore) was used for quantification of 32 chemokines and cytokines according to the manufacturer’s instructions. For Transforming growth factor beta 1 (TGF-β1), a DuoSet ELISA kit was used (R&D Systems) as described before [45]. For the analysis of the Luminex data all the values below the measured ranged were set to zero. IL-13 did not reach the lower limit of detection; therefore this cytokine was removed from the analysis. Data were scaled to unit variance prior to hierarchical clustering to account for different dynamic ranges of different cytokines. Hierarchical clustering using correlation as a distance measure and complete linkage was performed.

### RNA isolation and mRNA expression in FACS sorted cells

RNA was isolated from FACS sorted CD3, CD19 and CD11b cells after Trizol treatment.

cDNA was synthesized (input CD3: 25 pg, CD19: 10 pg and CD11b cells: 82 pg) using a Tetro cDNA synthesis kit (Bioline) according to the manufacturer’s instructions. qPCR was performed with SYBR Green (GC Biotech) on a Roche LightCycler. Primer sequences are listed in Supplementary Table 1. Targeted gene expression was related to the average expression of household genes HPRT, actin and GAPDH.

### Statistical analysis

To test statistical significance of differences in immune cell (CD45+, CD3+, B220+ and CD11b+) accumulation in prostates of aging mice of various genotypes and age of onset of adenocarcinoma of the prostate between Hi-Myc and Hi-Myc;RAG1^-/-^, linear regression analysis was applied. For comparison of infiltrating immune cell (CD45+, F4/80+) numbers and CXCL2, CCL5 and total TGF-β1 levels between genotypes at a specific age, unpaired, two tailed t-tests were applied. Analyses of data from Luminex cytokine assay were performed using public and in-house developed scripts in R statistical software 3.2.0 (https://www.r-project.org/). The concentrations of cytokines below the specified limit of detection were set to zero. Hierarchical clustering of the data was performed using Pearson correlation as the distance measure and compete linkage. Cytokines differentially expressed between HiMYC and HiMYCRAG mice were identified using Mann-Whitney test.

## SUPPLEMENTARY MATERIALS FIGURES AND TABLE



## Abbreviations

AR: androgen receptor
GEMM: genetically engineered mouse model
H&E: haematoxylin and eosin
HIV: human immunodeficiency virus
IACUC: institutional animal care and use committee/ DEC in Dutch dierexperimenten commissie
IF: immunofluorescence
IHC: immunohistochemistry
PIN: prostate intraepithelial neoplasia
TGF-β1: transforming growth factor beta 1
TRAMP: transgenic adenocarcinoma mouse prostate
WT: wild type
