# Supplementary material for: The adaptive immune system promotes initiation of prostate carcinogenesis in a human c-Myc transgenic mouse model
Source: Oncotarget. 2017 Sep 28;8(55):93867–77. doi: 10.18632/oncotarget.21305 (PMC5706841; doi:10.18632/oncotarget.21305)
Supplement: Supplementary file 1 [file oncotarget-08-93867-s001.pdf]

The adaptive immune system promotes initiation of prostate carcinogenesis in a human c-Myc transgenic mouse model

SUPPLEMENTARY MATERIALS

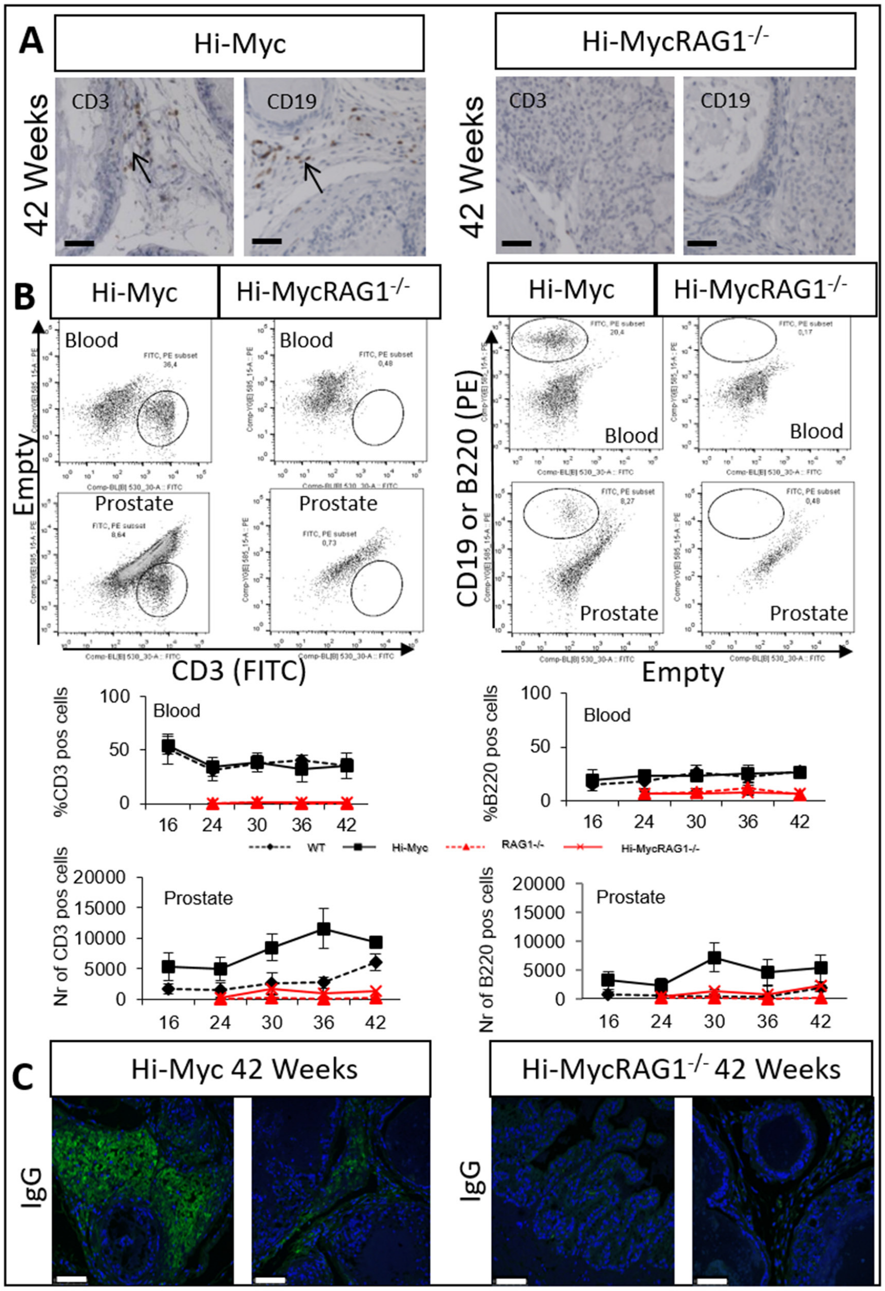

Supplementary Figure 1: Confirmation of absence of T and B cells in Hi-MycRAG1$^{-/-}$ mouse prostates. Hi-Myc and Hi-MycRAG1$^{-/-}$ mouse prostates and blood from 42 week old mice for **(A)** IHC staining for CD3 (T cells) and CD19 (B cells) (bar=100µM) **(B)** Flow cytometry analyses presented as dotblots and in graphs (n=5 error bars are SEM) and **(C)** Immuno fluorescent staining for IgG depositions (bar=75µM). Mature T and B cells are present in Hi-Myc mice but absent in Hi-MycRAG1$^{-/-}$ mice.

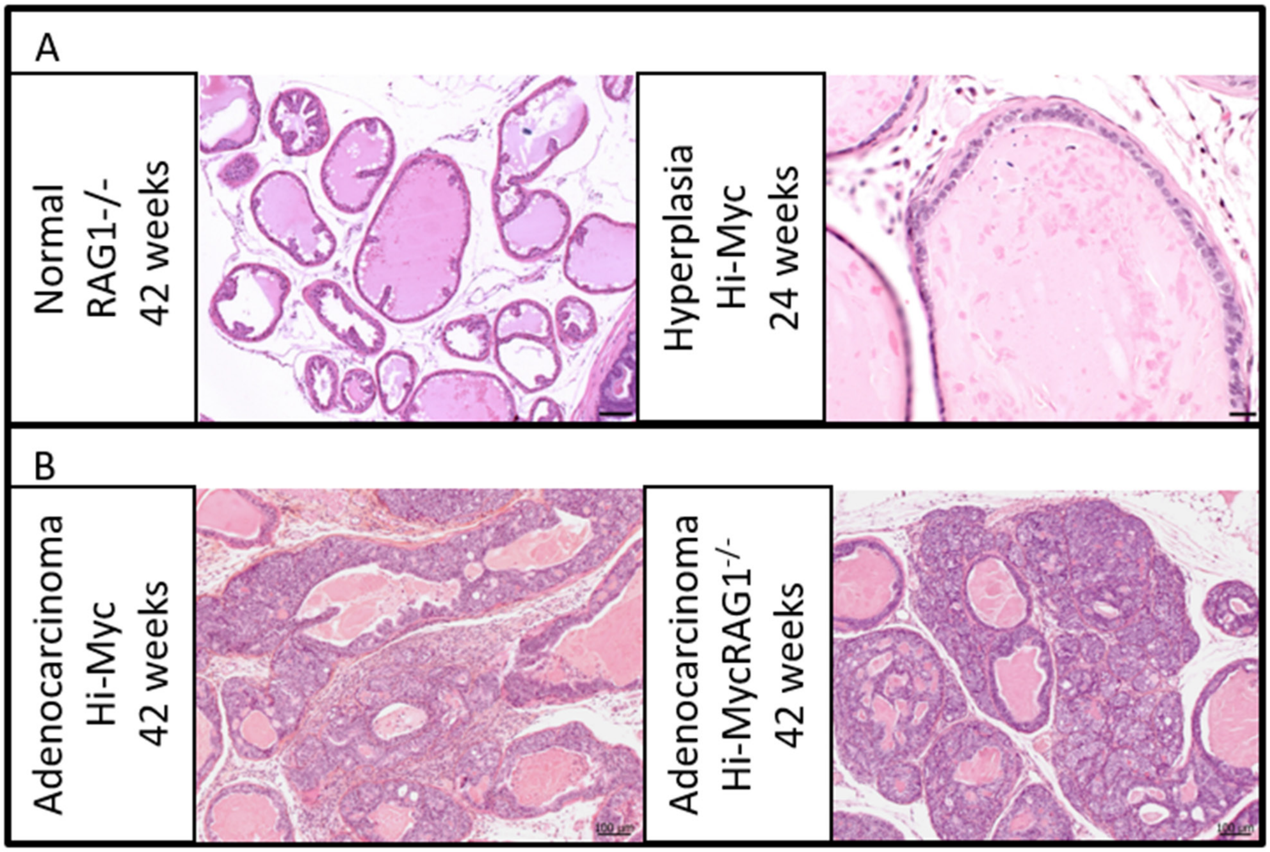

Supplementary Figure 2: Morphology of various stages of cancer development in mouse prostates. All H&E stainings. **(A)** Normal prostate tissue of a 42 weeks old RAG1$^{-/-}$ mouse (Left panel; bar 100µM) and hyperplasia in a 24 weeks old Hi-Myc mouse (Right panel; bar=20µM). **(B)** Adenocarcinoma in a 42 weeks old Hi-Myc mouse (Left panel) and Hi-MycRAG1$^{-/-}$ (Right panel; bar=100µM).

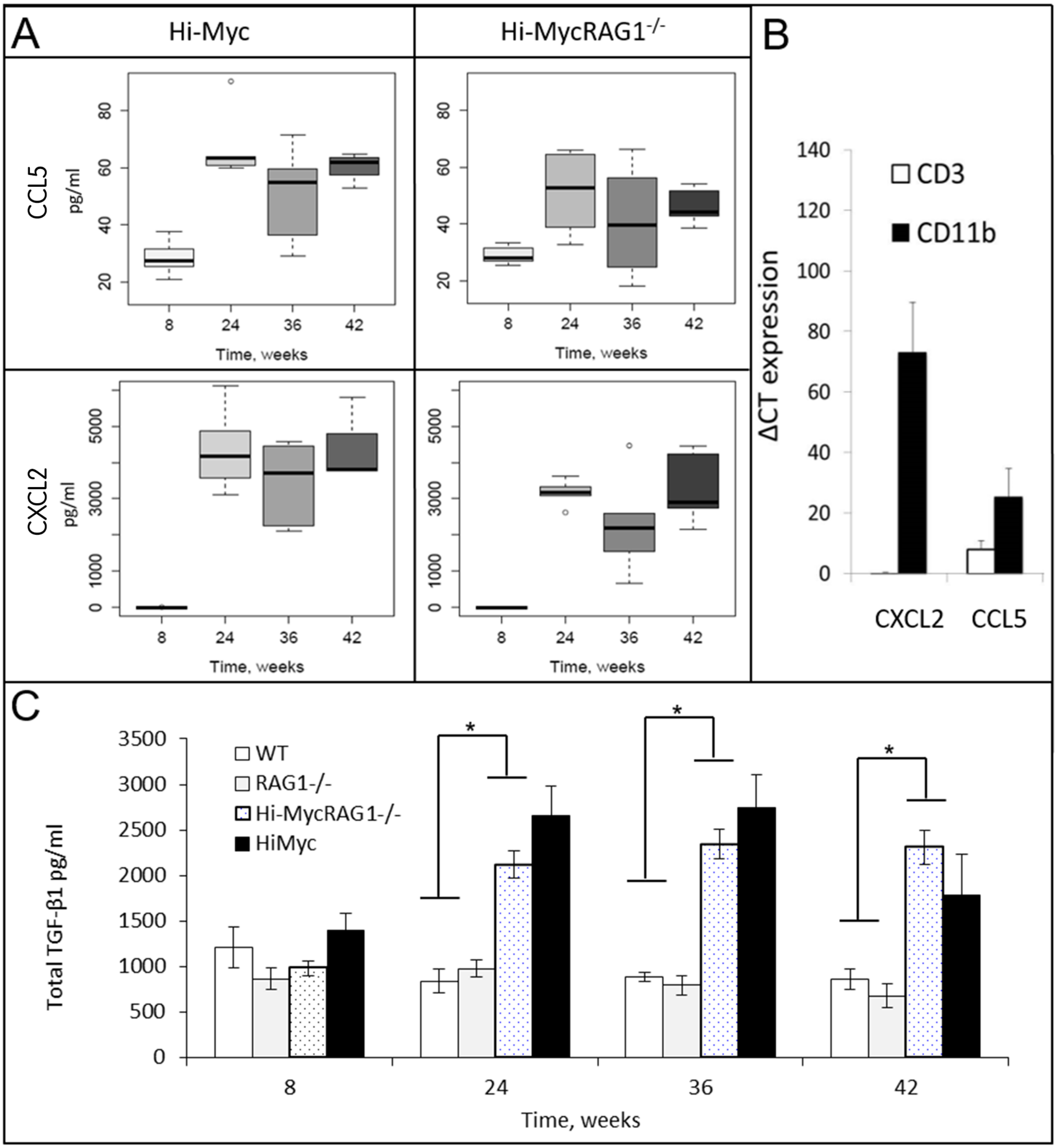

Supplementary Figure 3: CXCL2 and CCL5 and total TGF-β1 levels throughout prostate carcinogenesis. (A) Expression levels of CXCL2 and CCL5 in Hi-Myc and Hi-MycRAG1$^{-/-}$ prostates as assessed by Luminex array. Increased expression of both CXCL2 and CCL5 was observed in concert with prostate carcinogenesis (24-42 weeks) as compared to non-cancerous tissue (8 weeks) (n=5, $p<0.05$) **(B)** CXCL2 and CCL5 expression was measured by QPCR in CD3+ lymphocytes and CD11b+ myeloid cells isolated from the prostate associated lumbar lymph nodes and the prostate cancer microenvironment. CXCL2 was exclusively expressed by CD11b cells, while CCL5 was expressed by CD3 and CD11b cells. **(C)** Total TGF-β1 level was measured in prostate tissue using a DuoSet ELISA. Increased total TGF-β1 level was found in prostate cancer tissue (Hi-Myc and Hi-MycRAG1$^{-/-}$ of 24 weeks of age) compared to normal/ non-cancerous tissue (WT, RAG1$^{-/-}$ and 8 weeks Hi-Myc, Hi-MycRAG1$^{-/-}$) (n=5, *=$p<0.05$).

Supplementary Table 1: qPCR primer sequences

Gene name	Forward 5’ → 3'	Reverse 5’ → 3’
**CXCL2**	GAGCTTGAGTGTGACGCCCCCAGG	GTTAGCCTTGCCTTTGTTCAGTATC
**CCL5**	CCACTTCTTCTCTGGGTTGG	GTGCCCACGTCAAGGAGTAT
**Actin**	ACCAAGGTGTGATGGTGGGAATGG	GTAGATGGGCACAGTGTGGGTGAC
**GAPDH**	ACCACAGTCCATGCCATCAC	TCCACCACCCTGTTGCTGTA
**HPRT**	AGCTACTGTAATGATCAGTCAACG	AGAGGTCCTTTTCACCAGCA
